# Global atmospheric methane uptake by upland tree woody surfaces

**DOI:** 10.1038/s41586-024-07592-w

**Published:** 2024-07-24

**Authors:** Vincent Gauci, Sunitha Rao Pangala, Alexander Shenkin, Josep Barba, David Bastviken, Viviane Figueiredo, Carla Gomez, Alex Enrich-Prast, Emma Sayer, Tainá Stauffer, Bertie Welch, Dafydd Elias, Niall McNamara, Myles Allen, Yadvinder Malhi

**Affiliations:** 1 Birmingham Institute of Forest Research, University of Birmingham, Birmingham, UK; 2 School of Geography, Earth and Environmental Science, University of Birmingham, Birmingham, UK; 3 Lancaster Environment Centre, Lancaster University, Lancaster, UK; 4 School of Informatics, Computing and Cyber Systems, Northern Arizona University, Flagstaff, AZ USA; 5 CREAF, Cerdanyola del Vallès, Spain; 6 Department of Thematic Studies—Environmental Change, Linköping University, Linkoping, Sweden; 7 School of Environment, Earth and Ecosystem Studies, The Open University, Milton Keynes, UK; 8 Multiuser Unit of Environmental Analysis, University Federal of Rio de Janeiro, Rio de Janeiro, Brazil; 9 Institute of Marine Science, Federal University of São Paulo (IMar/UNIFESP), Santos, Brazil; 10 Smithsonian Tropical Research Institute, Balboa, Panama City, Republic of Panama; 11 Institute of Botany, Ulm University, Ulm, Germany; 12 UK Centre for Ecology & Hydrology, Lancaster Environment Centre, Lancaster, UK; 13 Environmental Change Institute, School of Geography and the Environment, University of Oxford, Oxford, UK; 14 Atmospheric, Oceanic and Planetary Physics, Department of Physics, University of Oxford, Oxford, UK; 15 Leverhulme Centre for Nature Recovery, University of Oxford, Oxford, UK

**Keywords:** Carbon cycle, Forest ecology, Carbon cycle

## Abstract

Methane is an important greenhouse gas^1^, but the role of trees in the methane budget remains uncertain^2^. Although it has been shown that wetland and some upland trees can emit soil-derived methane at the stem base^3,4^, it has also been suggested that upland trees can serve as a net sink for atmospheric methane^5,6^. Here we examine in situ woody surface methane exchange of upland tropical, temperate and boreal forest trees. We find that methane uptake on woody surfaces, in particular at and above about 2 m above the forest floor, can dominate the net ecosystem contribution of trees, resulting in a net tree methane sink. Stable carbon isotope measurement of methane in woody surface chamber air and process-level investigations on extracted wood cores are consistent with methanotrophy, suggesting a microbially mediated drawdown of methane on and in tree woody surfaces and tissues. By applying terrestrial laser scanning-derived allometry to quantify global forest tree woody surface area, a preliminary first estimate suggests that trees may contribute 24.6–49.9 Tg of atmospheric methane uptake globally. Our findings indicate that the climate benefits of tropical and temperate forest protection and reforestation may be greater than previously assumed.

## Abstract (web summary)

Studies of in situ woody surface methane exchange in upland tropical, temperate and boreal forest trees find that methane uptake can result in a net tree methane sink that is globally significant and demonstrates an additional climate benefit provided by trees.

## Main

Methane (CH_4_) is the most important anthropogenically enhanced greenhouse gas in the atmosphere after CO_2_, contributing an extra 26% of anthropogenic greenhouse warming since 1750 (ref. ^1^). As such, it is important that all sources and sinks are fully quantified to include the role of terrestrial ecosystems in mediating atmospheric exchange. The global CH_4_ budget is now unbalanced, with sources exceeding sinks, which leads to growth in the atmospheric concentration of this powerful greenhouse gas^2^. Soils represent an important CH_4_ sink in which CH_4_-consuming methanotrophs are ubiquitous^7^ but the collective CH_4_ uptake capacity of soils can vary, depending on a range of factors, including soil moisture and temperature^7–9^. At the global scale, the atmospheric CH_4_ sink terms (for example, hydroxyl (OH) radicals and ultraviolet-associated processes) dominate CH_4_ losses, with the far smaller soil sink term considered the only terrestrial sink. However, data-driven global modelling efforts tend to overestimate emission sources by about 151 Tg yr^−1^ when compared to smaller atmospheric ‘top-down’ derived estimates^2^, possibly suggesting that a substantial terrestrial CH_4_ sink term is either poorly quantified or missing from the global CH_4_ budget.

Recently, we showed that mature trees in saturated soils can emit substantial quantities of soil-produced methane^10–12^, which has been confirmed by others^13–15^. In the Amazon, for example, flooded trees are the single largest emission source from the region^3^. However, endophytic methane-using bacteria have been identified in temperate poplar trees^16–18^ and in tropical wetland tree bark, in which bark methanotrophy attenuated 36% of CH_4_ emissions^19^. Collectively, these lines of evidence raise the possibility that trees have the capacity not only to serve as an internal sink for otherwise emitted CH_4_ but also, where soil CH_4_ production is limited by low soil moisture, to serve as net sinks of atmospheric CH_4_.

Observations of tree CH_4_ uptake have primarily focused on trees in cool locations, in which flux rates in either direction tend to be low^5^; on trees growing in unusual and not necessarily representative landscapes^6^; or on wetland trees that have a known supply of soil-derived CH_4_, where emissions dominate^19^. However, some studies have reported the presence of negative CH_4_ fluxes on upland (non-flooded) tree stems, even though the main focus of these papers tended to be on net emissions^20^ (Supplementary Table 1). Indeed, measurement chamber positioning in the lowermost portions of the tree may have disproportionately identified emission fluxes over uptake, as the lowermost portions of tree stems are far closer to soil sources of CH_4_, which may be entrained by tree roots and then emitted to the atmosphere^4^ (Supplementary Table 1). Collectively, these studies do, however, present the possibility that if higher portions of upland tree stems and branches are considered, then trees on free-draining soils may be net sinks of atmospheric CH_4_. The net outcome of CH_4_ emission and uptake processes associated with tree surfaces would depend on the hydrological status of the soil and the methane-consuming capacity of the tree surface area of exchange. The large surface area of tree stems, branches and twigs^21^ (hereafter referred to as woody surfaces) and the relative abundance of upland (herein referring to trees on free-draining soils with low water tables) versus wetland forests means that even small fluxes, in either direction (emission and uptake), may result in large cumulative exchanges of CH_4_ associated with upland trees at the global scale.

To fully examine the role of upland trees in the global CH_4_ cycle, we examined in situ woody surface CH_4_ exchange spanning a latitudinal gradient of tropical, temperate and hemiboreal forest ecosystems. These measurements allowed us to quantify net exchanges of CH_4_ from woody surfaces across a range of biomes spanning tropical forests in Amazonia (Cuniã) and Panama (Gigante), temperate broadleaf forest in the United Kingdom (Wytham Woods) and hemiboreal coniferous forest in Sweden (Skogaryd). In addition, we made flux measurements from tree stems from three further locations in the Amazon floodplain during low water (the Negro, Solimões and Tapajós Rivers). These locations experience periodic inundation but also dry periods when the water table is many metres below the soil surface^19^.

## Global tree stem flux measurements

**Fig. 1:**
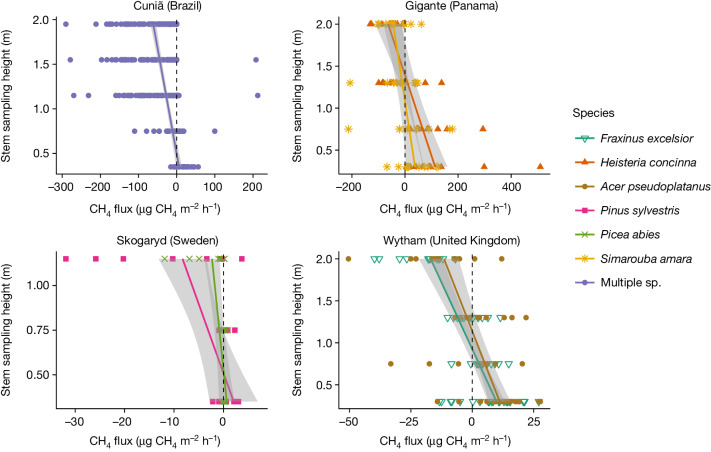
Regression plots of methane (CH_4_) fluxes against tree stem sampling position above the forest floor. Top left, fluxes measured from Cuniã, Brazil, from two free-draining forested plots (20 × 30 m^2^) in the catchment draining to the Madeira River (*n* = 100) during 15–25 March 2013; top right, for mature trees stems of two common tree species: *Heisteria concinna* (orange) and *Simarouba amara* (yellow) in experimental litter manipulation treatments in a tropical forest on free-draining soil in Panama, Central America, between 18 and 27 November 2015 (24 trees); bottom right, ash and sycamore in a temperate deciduous woodland on free-draining soil in Wytham Woods, United Kingdom, between October 2015 and January 2016 (24 trees); bottom left, measurements from Scots pine and Norway spruce (18 trees) in the Skogaryd Research Catchment, near Vänersborg in southwestern Sweden. Grey bands denote 95% confidence intervals, which for Cuniã (large number of trees) falls in the regression line area. In Cuniã (Brazil) and Gigante (Panama) upland forests, CH_4_ uptake fluxes were largest at the highest sampling points at about 1.75–2 m above the forest floor (−56.5 ± 59.4 µg of CH_4_ m^−2 ^h^−1^ and −46.7 ± 64.7 µg of CH_4_ m^−2 ^h^−1^, respectively). For figure clarity, some of the largest values are not included, but the highest individual measured uptake values were −290 µg of CH_4_ m^−2 ^h^−1^ in Cuniã and −488 µg of CH_4_ m^−2 ^h^−1^ in Gigante. At Wytham, we found mean uptake at the highest sampling position (2 m) of −18.5 ± 20.4 µg of CH_4_ m^−2 ^h^−1^ for ash and −14.0 ± 27.4 µg of CH_4_ m^−2 ^h^−1^ for sycamore, with the highest recorded individual uptake fluxes being −54.8 µg of CH_4_ m^−2^ h^−1^ on ash and −142 µg of CH_4_ m^−2^ h^−1^ on sycamore. General information relating to these sites is presented in Extended Data Table 5. Source Data

We examined CH_4_ fluxes, in either direction, across several tree stem heights using established methods^22^ that couple rapidly deployable woody surface gas exchange chambers with laser-based analysers. In addition, for two sites (Cuniã in Brazil and Skogaryd in Sweden) we collected tree stem wood cores to evaluate vertical trends in methanotrophy with height above the forest floor and assessed stable carbon isotope values of CH_4_ (δ^13^C-CH_4_ values) from within-chamber air. The CH_4_ uptake fluxes were largest in tropical forests and smallest in temperate and hemiboreal sites. Despite high flux variability, trees in each of the four measured upland sites spanning the latitudinal gradient consistently exhibited a vertical pattern of CH_4_ exchange. CH_4_ emissions were observed in the lowermost portions of the tree stem (about 30–130 cm; Fig. 1), and fluxes tended to switch from emission to CH_4_ uptake at and above breast height (1.3 m), although we observed uptake starting lower down the tree stem (0.7 m) in Cuniã, Brazil (Fig. 1). At our temperate site at Wytham Woods (United Kingdom), the same pattern was observed for two dominant tree species, ash and sycamore. We further measured CH_4_ uptake from branches in oak tree crowns at 11 m above the forest floor in Wytham Woods on a single occasion during a prolonged dry period in June 2018, where we found net CH_4_ uptake (−1.86 ± 1.89 µg of CH_4_ m^−2 ^h^−1^, *n* = 11). Although small and highly variable from this limited sampling, it confirms, along with previous work on Swedish branches^17^, that uptake processes also take place on woody surfaces higher up in the tree and water limitation may influence the size of uptake processes.

**Fig. 2:**
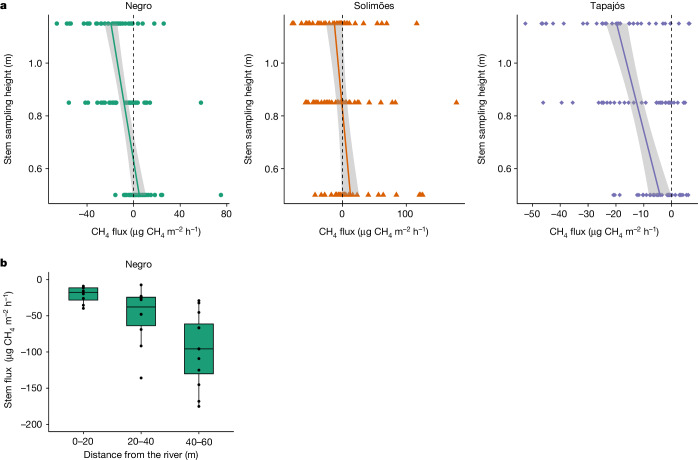
Spatial patterns of CH_4_ fluxes on dry season Amazon floodplain trees. **a**, Regression plots of CH_4_ fluxes against tree stem sampling position. Measured from above the forest floor in three floodplain locations during low water in the central Amazonian floodplain (36 trees from each plot). **b**, Fluxes measured at 5 m above the forest floor with increasing horizontal distance from the Negro River (*n* = 36 trees). Source Data

At the coolest location, hemiboreal forest in Skogaryd (Sweden), the highest sampling position was located 115 cm above the forest floor and fluxes were correspondingly small, although uptakes of −9.90 and −2.82 µg of CH_4_ m^−2^ h^−1^ were observed for pine and spruce tree stems, with a declining pattern of emission favouring uptake with increasing height above the soil surface. Although fluxes across all biomes tended to be highly variable, our study design, along with many trees examined in some locations (for example, Cuniã), allowed us to detect general patterns. These tree CH_4_ uptake patterns are further supported by observations in the three Amazonian floodplain locations. Here, trees were a significant source of CH_4_ during periods of inundation^19^. However, during low water-table conditions in the dry season (water table about 6–10 m below the soil surface), trees demonstrated an identical pattern of emission in the lowermost stem portions (Negro and Solimões Rivers) and then uptake from 60 cm (Negro River) to 90 cm (Solimões River) above the forest floor (Fig. 2a). In the location with the lowest water table, Tapajós, we observed mainly uptake at all sampling positions above the forest floor, in common with observations in Cuniã. These floodplain trees therefore demonstrate plasticity in their trace gas exchange capacity, with the hydrologically contingent capability to function as both large, yet spatially constrained point sources of atmospheric CH_4_ or sinks representing far more spatially extensive upland forest, depending on soil water-table level.

## Methane oxidation potential in trees and its isotopic enrichment

Incubations of wood cores from Cuniã in Brazil and Skogaryd in Sweden yielded a consistent capacity for CH_4_ uptake in response to either ambient CH_4_ concentrations (high-affinity methanotrophy—MOP_HA_) higher up the tree stem or enriched CH_4_ concentrations (low-affinity methanotrophy—MOP_LA_) at the stem base (Extended Data Table 1). We attribute this pattern to a supply of soil-derived higher-than-ambient CH_4_ near stem bases^16^, sustaining a larger population of low-affinity methanotrophs than higher up the tree. By contrast, high-affinity CH_4_ oxidation dominates higher up the tree stem (Extended Data Table 1), in which CH_4_ is supplied at ambient atmospheric concentrations. Thus, the observed pattern of CH_4_ emissions (or low uptake) in the lowermost portion of tree stems (less than 1 m) and substantial uptake higher up the tree stem are consistent with expected methanotroph populations occupying niches that are determined by CH_4_ supply. Further, δ^13^C-CH_4_ analyses from floodplain trees in Central Amazonia during an exceptional dry season demonstrate strong enrichment during chamber incubations on woody surfaces at 5 m above the forest floor. Less ^13^C enrichment in CH_4_ was found for soil uptake at those locations (Extended Data Table 2) pointing to a much stronger atmospheric CH_4_ oxidation potential by methanotrophic bacteria on the tree surfaces.

Bringing these lines of evidence together demonstrates a highly variable but near-ubiquitous capacity for tree woody surfaces to remove atmospheric CH_4_ at ambient concentrations, with vertical spatial patterns of CH_4_ exchange corresponding to distance from the source of any soil-produced CH_4_. We observed, across all biomes, a decline in CH_4_ emission with distance from the forest floor, followed by a switch to uptake above a certain point at or around breast height (100–130 cm above the forest floor), or lower in the Amazon region. This is further reinforced by our measurements from trees in which larger net atmospheric CH_4_ uptake was observed at 5 m above the forest floor with increasing horizontal distance from the river: a probable response to progressively deeper water table with increasing distance from the river and a correspondingly smaller soil CH_4_ source (Fig. 2b). The CH_4_ oxidation potential observations provide a mechanistic explanation of this spatial pattern, as supported by recent findings of methanotrophs in wetland tree bark^16^, in combination with declining diffusive loss of soil-derived CH_4_ to the atmosphere with height above the forest floor and strong δ^13^C-CH_4_ enrichment on woody surfaces at height above the forest floor (Extended Data Table 2).

## Global importance of tree CH_4_ uptake

**Fig. 3:**
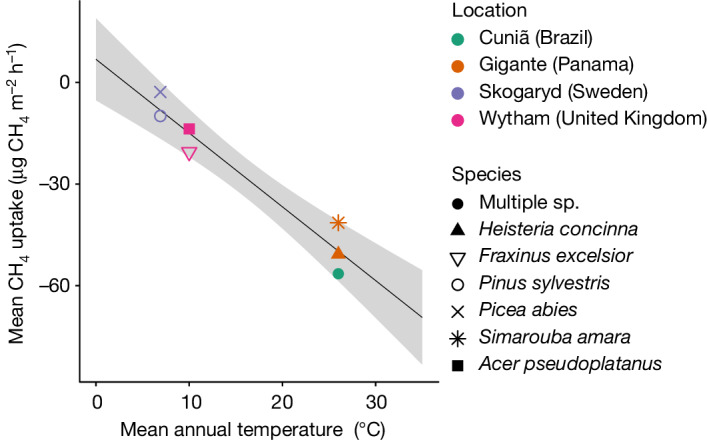
Empirical model of CH_4_ uptake versus MAT. Mean CH_4_ uptake recorded in the uppermost tree sampling position above the forest floor in each of the upland forest locations, plotted against MAT for each site. The regression equation is CH_4_ uptake = −2.179 MAT + 6.837. The grey shading represents the 95% confidence interval. Source Data

There are complex interactions between vegetation and the atmosphere with respect to CH_4_ exchange. Wetland trees tend to be large sources of CH_4_ (refs. ^12,19^), and these sources are well accounted for in the global CH_4_ budget. Although there is evidence to suggest widespread aerobic CH_4_ production in living organisms through reactive oxygen species^23^, aerobic emissions from foliage are thought to be small (less than 1 Tg yr^−1^)^24^, although uncertainties are large. They further conflict with the recent finding of uptake in some forest canopy leaves^25^, although this has yet to be replicated. Our findings do provide compelling evidence that woody surfaces in upland forests are net sinks in the global CH_4_ budget. We therefore assessed the global importance of this woody surface net atmospheric CH_4_ sink term in the Earth system in two steps. First, we examined how the mean CH_4_ uptake in the uppermost tree stem sampling positions (2 m, across our climate gradient) varied with mean annual temperature (MAT), which showed a strong relationship with larger uptake fluxes at higher MAT (Fig. 3). Given that we either sampled from large numbers of trees for which species identification was difficult (Amazonia *n* = 100) or from dominant tree species in established and well-monitored plots (Panama, Wytham and Skogaryd) and that we made several measurements on each tree at several positions above the forest floor, we consider our individual site data to be representative, thus permitting large-scale analysis and interpretation. We also consider our approach to be conservative because we only included the mean uptake values at the highest point that we measured on the tree stem, no higher than 2 m above the forest floor, in our global regression with mean MAT. However, our regression analysis of CH_4_ uptake versus tree stem sampling position suggests the probable presence of larger uptake values, on average, higher up the stem, which is supported by our measurements at 5 m above the forest floor in the Amazon floodplain during an exceptional dry season, which show twice the CH_4_ uptake (about 100 µg of CH_4_ m^−2^ h^−1^; Fig. 2b) of those measured at 2 m above the forest floor elsewhere in the tropics (Figs. 1 and 3). Second, we sought to quantify the global woody surface area. Until recently, reliable estimates of tree woody surface area have been limited. We used new terrestrial laser scanning (TLS) approaches to measure woody surface area in each of the temperate and tropical sample biomes and apply those to forests globally.

We then scaled estimated CH_4_ uptake across the globe based on tree canopy cover and mean climatology. Finally, we aggregated the CH_4_ uptake across biomes to determine per-biome values (Extended Data Table 3). A first approach that excludes any CH_4_ uptake from woody surfaces more than 4 m above the forest floor suggests a highly conservative uptake estimate of 1.7 Tg of CH_4_ yr^−1^. However, considering all woody tree surfaces leads to a mean total global woody surface CH_4_ sink estimate of 37.2 Tg of CH_4_ yr^−1^ (24.6–49.9 Tg of CH_4_ yr^−1^), with the range demonstrating uncertainty associated with the land surface products used, which offer very different global woody surface area estimates (Extended Data Table 4) or whether woody shrubs are included (‘high’ estimate, Extended Data Table 3). Despite this uncertainty, the size of this global CH_4_ woody surface uptake is similar to that of the global soils sink (30–40 Tg of CH_4_ yr^−1^)^2^, thus far the only other characterized terrestrial CH_4_ sink in the Earth system. We estimate the total global aboveground woody surface area to be 143 (±59) million km^2^ (Extended Data Table 4), approximately equal to total global land surface area (149 million km^2^), but, unlike the global land (soil) surface, this area of CH_4_ exchange will vary with changing forest cover. Hence, woody surface extent and architecture adds a poorly appreciated structural third dimension for exchange between the land biosphere and atmosphere.

## Implications and conclusions

We show that woody surfaces of most trees constitute a new sink term for CH_4_, which presents opportunities to close the now unbalanced global CH_4_ budget. In this budget, fully integrative top-down estimates of atmospheric CH_4_ removal have been smaller than bottom-up estimates, which have neglected the process we now uncover^2^. Further, our findings indicate that there may be substantial CH_4_-associated climate benefit from the presence of trees, which is not now accounted for in natural climate solutions frameworks. Indeed, as the net annual C sequestration of tropical rainforests declines^26^, the contribution of this upland tree woody surface CH_4_ sink term may increase in relative importance because it probably responds to increasing temperature and atmospheric CH_4_ concentration^5^.

Further, the woody surface tree uptake mechanism shows opportunities for enhanced mitigation of atmospheric CH_4_ growth. The largest biomes contributing to this total woody methane sink are tropical and subtropical moist broadleaf forests (16.5–22.7 Tg of CH_4_ yr^−1^ for our ‘low’ and ‘high’ estimates; Extended Data Table 3) and tropical and subtropical grasslands, savannas and shrublands (0–14.3 Tg CH_4_ yr^−1^ for our low and high estimates; Extended Data Table 3). To first estimate the methane-associated climate benefit of long-term intact forests, we equate a 1 Mg of CH_4_ yr^−1^ constant methane sink with removal of 8 Mg of CO_2_-warming equivalent (we) yr^−1^ (ref. ^27^). For a mature Amazonian forest, we therefore estimate that its tree stem surface CH_4_ sink is equivalent (in terms of the equivalent amount of C in CO_2_) to 0.037 Mg of CO_2_-C ha^−1^ yr^−1^, approximately 15% the value of the mean biomass carbon sink in Amazonian forests (about 0.24 Mg of C ha^−1^ yr^−1^; ref. ^26^), thus pointing to a significant extra climate benefit of intact tropical forest biomes and probable benefit to large-scale forest regrowth and restoration

We consider the CH_4_ sink consequences of removing trees to be small relative to total biomass C loss; however, the impact of reforestation may be more significant. Despite the lower biomass of secondary forests, their large numbers of small trees mean that they often have high woody surface area, similar to or higher than that of old growth forests^28,29^. We estimate an extra greenhouse gas mitigation value from CH_4_ uptake as equivalent to 0.131 and 0.586 Mg of C ha^−1^ yr^−1^ in temperate and tropical forests, respectively, corresponding to a 7% and 12% extra climate benefit of new trees in these respective biomes. This suggests a possible global extra climate benefit, through the enlarged tree CH_4_ sink, equivalent to up to 0.3 Pg of C yr^−1^ or 1.1 Pg of CO_2_-we yr^−1^. This is equivalent to a 10% extra mitigation potential over benefits already estimated for expansion of temperate and tropical forests^30,31^. Hence, the tree CH_4_ sink may have a particularly important climate mitigation role in the context of reforestation, though this prediction needs to be tested through field studies in regrowing forests.

Our findings demonstrate high spatial and taxonomic variability in the CH_4_ uptake capacity of trees. Identifying tree species with the largest capacity for CH_4_ uptake offers opportunities to further address the growth in atmospheric CH_4_. Measurements along the full vertical profile of trees may find a yet stronger CH_4_ sink in tree branches.

## Methods

**Extended Data Fig. 1:**
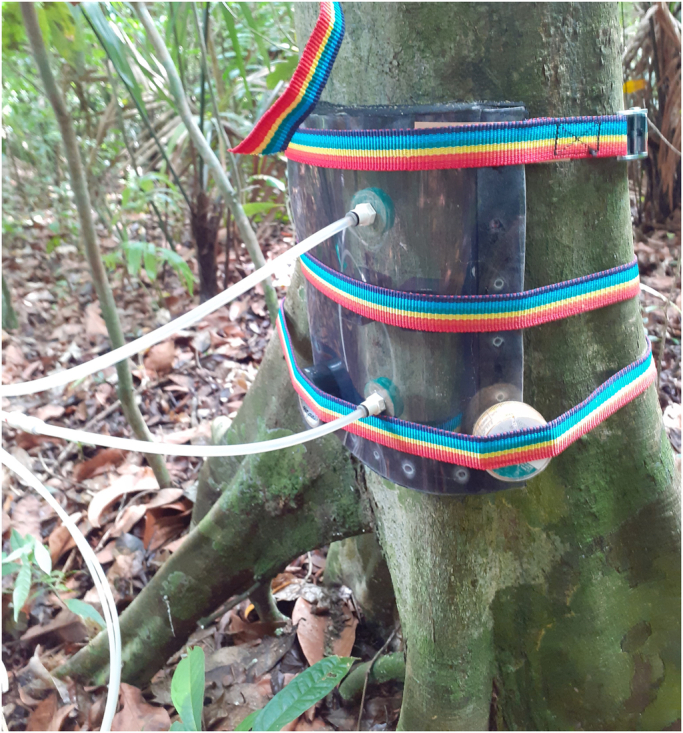
Photograph of a typical tree woody surface flux measurement chamber. Each chamber has an inlet and an outlet that join to a laser methane analyser. See methods. For a fuller description of the method see Siegenthaler et al.^22^. Photograph by Josep Barba.

Individual tree CH_4_ flux observations were made as follows in each of the following sites. Chambers used at all sites were constructed from gas-impermeable polyethylene terephthalate or polycarbonate plastic sheet. The chamber characteristics, accuracy and precision of the method when used either in manual syringe mode for later analysis on Los Gatos analysers or in continuous mode (with in situ analysis by means of field portable microportable and ultraportable greenhouse gas Los Gatos analysers (MGGA and UGGA, respectively)) are detailed in ref. ^22^. To summarize, and unless specified below, two ‘sleeve’ chamber sizes were used, small (25 × 16 × 1.5 cm^3^) and large (30 × 24 × 1.5 cm^3^), depending on the dimensions of the woody surface being measured. All fluxes were measured in the middle part of the day (10:00–15:00) or at times between 09:00 and 18:00. Fluxes measured in continuous flow mode had variable deployment and measurement periods, depending on the rate of concentration change, which was observed in real time. A photograph of a typical chamber deployment is presented in Extended Data Fig. 1. The minimum flux that could be detected using the modified fast methane analyser (FMA) analysis method^32^ based on instrument sensitivity and chamber volume was 0.4–3.5 µg of CH_4_ m^2^ h^−1^. The minimum flux that could be detected using the LGR UGGA and the LGR MGGA in real-time measurements based, respectively, on instrument sensitivities of 4 ppb with 1 s precision and 2 ppb with 1 s precision, as well as on chamber volume, is less than 1 µg of CH_4_ m^2^ h^−1^.

### Cuniã Nature Reserve, Amazonia, Brazil (63° 5′ W, 8° 1′ S)

Stem CH_4_ emissions from mature trees (equal to or more than 10 cm diameter) were measured from two free-draining forested plots. The two plots (20 × 30 m^2^) were located in the Madeira River catchment, a white-water river system and one of the largest tributaries of the Amazon. The Madeira drains the Andean area upstream, which results in high suspended and dissolved solids concentrations in water, with neutral to alkaline pH^33,34^. The MAT is 26 °C and mean annual rainfall is 2,500 mm (ref. ^35^).

Methane flux measurements from mature trees stems (*n* = 50 per plot) at five stem heights (20, 60, 100, 140 and 180 cm above the soil surface) were performed during a period of transition towards the dry season when the water tables in both the plots were more than 10 m below the soil surface. Measurements were carried out between 15 and 25 March 2013. Methane fluxes were measured using static chambers as described in refs. ^3,22^, with air samples from the static flux chambers drawn using 30 ml syringes and immediately transferred to a 12 ml exetainer (Exetainer) for later analysis of CH_4_ using the modified LGR CH_4_ laser-based analyser^3^ (LGR UGGA).

### Gigante Peninsula, Barro Colorado Nature Monument, Panama (9° 6′ N, 79° 54′ W)

Measurements in semi-evergreen tropical forest were carried out between 18 and 27 November 2015 in the five control plots of the Gigante Litter Manipulation Project, approximately 5 km south of Barro Colorado Island, Panama, Central America. A full description of the litter manipulation experiment is given in refs. ^36,37^. The MAT at the weather station on Barro Colorado Island is 26 °C, mean annual rainfall is 2,600 mm and there is a strong dry season from mid-December to mid-April^38^.

We measured tree stem CH_4_ fluxes at 30, 75, 130 and 200 cm height from two common tree species: the fast-growing canopy tree *Simarouba amara* (Aubl.) and the shade-tolerant subcanopy tree *Heisteria concinna* (Standl.) (12 trees per species). Tree stem gas fluxes were measured using a flexible chamber (45 cm × 30 cm × 19 mm polycarbonate) as described in refs. ^4,20^. Gas samples were taken by syringe from a septum in the middle of the chamber at 0, 5, 10 and 15 min and injected into pre-evacuated 12 ml borosilicate vials. All samples were analysed in the United Kingdom using off-axis integrated cavity output spectroscopy (FMA-200 fast methane analyser).

### Wytham Woods, Oxfordshire, United Kingdom (51° 46′ 42″ N, 1° 19′ 42″ W)

Measurements at the temperate site were conducted in four control plots of a litter manipulation experiment^39,40^ at Wytham Woods, an old growth (about 120 yr) mixed deciduous woodland in Oxfordshire, United Kingdom. The canopy at the study site is dominated by ash (*Fraxinus excelsior* L.), beech (*Fagus sylvatica* L.), sycamore (*Acer pseudoplatanus* L.) and oak (*Quercus robur* L.)^41^. MAT was 10 °C (ref. ^40^). In each 25 × 25 m^2^ plot, three individuals each of ash and sycamore were randomly selected, making a total of 24 trees. Tree stem CH_4_ fluxes were sampled using the same chamber design, sampling heights and procedure as described for Gigante above, except that gas samples were collected at 0, 3, 6 and 10 min. All samples were analysed in the United Kingdom using off-axis integrated cavity output spectroscopy (FMA-200 fast methane analyser).

### Skogaryd, Sweden

The stem and soil flux measurements were conducted in the Skogaryd Research Catchment, near Vänersborg in southwestern Sweden, on three occasions in spring 2014 (2–3 April, 14–16 April and 28–30 April). Tree stem CH_4_ fluxes were measured from Norway spruce (*Picea abies* (L.) Karst.) and Scots pine (*Pinus sylvestris* (L.)), based on the dominance of these tree species in the European boreal forest^42^ and at the study site. Methane fluxes from tree stems were measured in real time using static chambers connected to a laser-based CH_4_ analyser (LGR UGGA) as described in ref. ^3^. Stem CH_4_ emissions were measured from mature tree stems (equal to or more than 10 cm, *n* = 9 per species) from three stem heights (20, 60 and 100 cm above the soil surface). The water table was more than 5 m below the surface in the plot and soil was characterized as a histosol. The mean air temperatures during the three sampling occasions were 13.1, 14.2 and 18.1 °C, respectively, with all measurements carried out during daytime (09:00–18:00). MAT is 6.9 °C.

Following stem flux measurements at the Skogaryd and Cuniã study sites, we assessed the CH_4_ oxidation potentials (both high affinity and low affinity) in the tree stems by extracting wood cores at 30 and 130 cm stem height from a subset of the trees (Cuniã, 80 trees; Skogaryd, 9 trees per species).

Wood cores were extracted at four cross-sections radially from bark to pith at the same location on the stem where CH_4_ flux measurement were performed using a 5.1 mm increment corer. These cores were immediately transferred into a 50 ml vial and incubated on the same day of sampling to quantify the potential rates of CH_4_ production and oxidation. Incubations were carried out onsite in the dark, at field temperature of 25–27 °C and 11–15 °C, respectively. An initial headspace CH_4_ concentration of 6.5 and 750 ppm was maintained for high- and low-affinity CH_4_ oxidation potentials, respectively, and the cores were incubated aerobically for 48 h. Headspace samples from all incubations were extracted at 4, 24 and 48 h and CH_4_ concentrations analysed using methods described in refs. ^3,12^.

### Further measurements made in the Amazon floodplain

Sampling design and measurement protocols for fluxes presented in Fig. 2 are detailed in refs. ^19,20^. In summary, we established three temporary plots (60 × 60 m^2^) in the floodplains of three principal rivers of the Amazon, the Negro (black water), Solimões (white water) and Tapajós (clear water). The CH_4_ fluxes were measured from a total of 108 trees (36 across each plot) at vertical intervals above the forest floor during low water in January 2018. We returned in the exceptional dry season of 2021 (October) to make measurements from a subset of trees at 5 m above the forest floor and also to sample for methane isotopes.

#### Chamber CH_4_ isotopes in the Amazon

For δ^13^C-CH_4_ analysis, 30 ml gas samples were collected from tree woody surfaces and soil surfaces at the Negro and Solimões floodplain forests during the dry season of 2021. Samples were taken from air and from flux chambers on the soils surface and tree stem surface at 5 m above the forest floor using gas-tight syringes and then transferred to pre-evacuated 12 ml borosilicate vials fitted with double wadded caps (Exetainer). Vials were over-pressurized to prevent ingress of air from pressure or temperature changes during transport to the laboratory. The δ^13^C values of CH_4_ were analysed using a cavity ring-down spectrometer (model G2201-i, Picarro) coupled with a custom-built auto-sampler and are reported relative to the Vienna Pee Dee Belemnite standard. The instrument was calibrated for δ^13^C-CH_4_ using isotopic reference gases with isotope ratios of −23.9‰, −54.5‰ and −66.5‰ (Isometric Instruments). The overall analytical precision based on replicate measurements of reference gases was ±0.4‰.

#### CH_4_ uptake global estimate methods

Our study leveraged TLS technology to develop a new surface area allometry^43^. TLS provides high-resolution, three-dimensional representations of tree structures, enabling precise surface area estimations. We scanned a total of 2,161 trees across 22 plots in tropical forests, temperate conifer forests, temperate broadleaf forests, temperate dry eucalypt forests and tropical savannahs to capture a wide range of tree morphologies. We built two woody area index (WAI) allometries (one for tropical forests, one for the rest of the world) to predict woody surface area from tree stem diameter (diameter at breast height, DBH) based on three-dimensional models of trees^43^. Each tree was scanned from several angles to ensure comprehensive coverage and cylindrical models were fit with TreeQSM^44^. These models were analysed using the treestruct R package^45^, which resulted in woody surface area and DBH for each tree. We then applied hierarchical generalized additive models^46^ to build the surface area allometric equations. These models were designed to capture the complex, nonlinear relationships between tree surface areas and their respective DBH (Extended Data Fig. 3).

To scale our TLS-derived allometry to forest plots, we integrated tree census data from 70 global ecosystem monitoring plots that were chosen to have close to 100% canopy cover and be generally greater than 5 m tall. Six Centre for Tropical Forest Science plots across the tropics were also used, which, in addition to their greater than 10 cm census, included trees down to 1 cm diameter. In most plots, trees of more than 10 cm DBH were measured, and we used plots for which trees were measured down to more than 1 cm DBH (six ForestGeo plots^47^) to estimate the percentage contribution of trees with 1–10 cm DBH to total woody surface area. We applied our allometry to each tree in these plots, thereby estimating the total woody surface area for each plot. We then performed a weighted average, based on plot size, across all plots to determine mean surface area for tropical forests.

The integration of TLS data with forest plot census data not only validated our allometric model but also allowed the extrapolation of surface area estimates across different biomes and forest structures.

#### Global scaling using satellite remote sensing data

For global-scale extrapolation, we used a combination of satellite remote sensing datasets. This approach allowed us to estimate woody surface areas across the world’s ecosystems, integrating our allometric model with global forest cover data.

We used The Nature Conservancy’s Ecoregions map to determine biome extents and both the 1 km global consensus land cover project^48^ and the MODIS MOD44B Version 6 Vegetation Continuous Fields (VCF^49^) 2019 product, as well as the 1 km consensus land cover map, to determine per-pixel forest cover. This dataset provided a global view of vegetation cover, quantified on a continuous scale for each pixel.

Combining these datasets, we were able to scale our allometric model from individual trees to a global scale. For each pixel, we determine the per-hectare woody surface area based on the undisturbed forest plots in that ecoregion and applying our allometry-derived WAI. We then scaled (multiplied) that potential value by the proportion of that pixel covered by forest, according to the 1 km consensus map and MODIS VCF.

To account for climatic variations in our model, we used the ERA5 monthly climate dataset^50^. This dataset provides mean monthly temperatures, which we converted to MAT. With the woody surface area in each pixel known (Extended Data Table 4), we then applied the CH_4_ uptake versus MAT regression across the globe (Extended Data Table 4).

**Extended Data Fig. 2:**
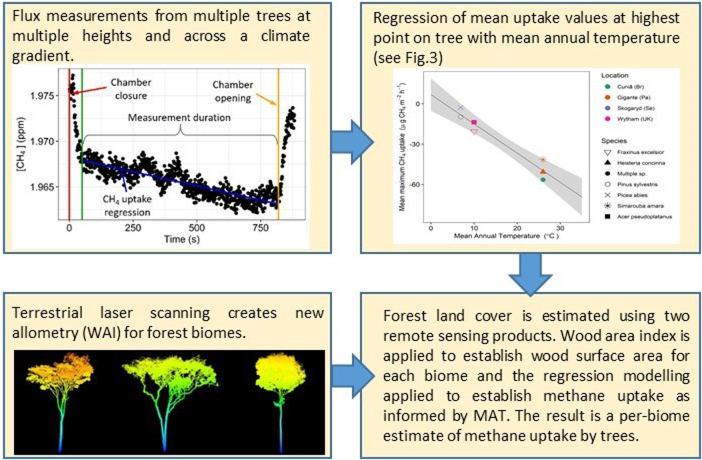
Schematic workflow diagram. Diagram of approach to deriving global, per-biome woody surface methane uptake estimates.

**Extended Data Fig. 3:**
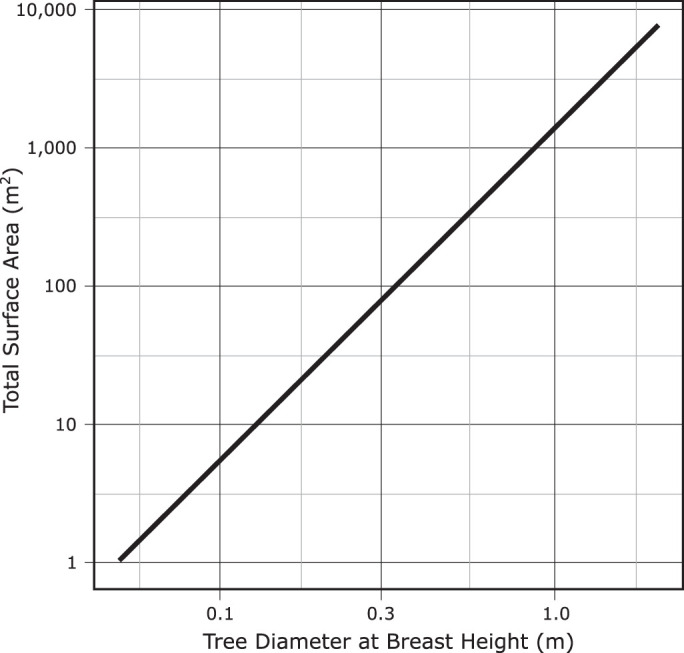
Woody surface allometry. Allometric relationship between tree woody surface area and diameter at breast height.

Finally, we aggregate the CH_4_ uptake across biomes to determine per-biome figures (Extended Data Table 3). The entire approach is summarized in Extended Data Fig. 2.

#### Global upscaling uncertainty analysis

We examined how woody surface area was influenced by various parameters, in particular the branch size fraction. Small branches and twigs carry greater uncertainty in our scaling because of challenges in estimation of their area from TLS measurements. Exclusion of twigs smaller than 2 cm in diameter from our analysis resulted in a 30% reduction in calculated uptake at the biome level and exclusion of branches smaller than 5 cm in diameter resulted in a 61% reduction in uptake (Extended Data Table 6). This represents uncertainty in our biome and global-scaled estimates, which may be reduced in future with more measurements in these smaller branch size fractions and better estimation of their surface area.

A second source of uncertainty is the relatively low uptake flux estimates derived from tropical trees at 2 m above the forest floor for our upscaling estimates, as opposed to fluxes that were twice as large when measured at 5 m (Fig. 2b), which is likely to counterbalance any such reductions from excluding small branch area size fractions. Because of the limited size of our dataset at 5 m height, we chose to use only the smaller 2 m flux values for our scaling. If the 5 m values are more typical of the whole tree, which seems plausible as most of the tree surface area is above 5 m and further away from any soil-generated methane carried through and lost from lower portions of the tree trunk, then our biome-scaled fluxes would increase by up to 100%. Hence the possible biases in small branch fluxes and flux sampling height probably work in opposite directions and cancel each other out to some extent. These uncertainties can only be reduced by a greatly expanded series of measurements of woody surface methane measurements in tropical trees at a range of heights and branch sizes, coupled with fine-scale assessment of small branch surface area.

We further considered forest structure uncertainty introduced through the WAI allometry. We considered variability in TLS-derived WAI across the 60 census plots spanning the tropics, which were used to inform the metric. The weighted mean of the surface area per hectare is 41,176 m^2^ (the WAI of 4.12 we applied to our estimates). Using Cochrane’s^51^ formula for variance of a weighted mean, we identify an s.e. of 221 m^2^ with the 95% confidence interval (1.96 × s.e.) of 433 m^2^ on either side of the mean, which, when propagated across our upscaling approach, falls well within the broader uncertainties already detailed.

A further uncertainty concerns local hydrological or humidity control of woody surface methanotrophy functioning. We have therefore eliminated water-limited biomes from our low estimates (Extended Data Table 3) and so provide a representative estimate spread that takes into account this uncertainty. Finally, there is some variability in the CH_4_ exchange behaviour of floodplain trees with respect to hydrology. They act as large point sources of CH_4_ when inundated, contributing to the comparatively well-known global wetland CH_4_ source, but our data show they also take up CH_4_ during the dry season, albeit with orders of magnitude smaller fluxes. Given the small area of tropical floodplain forests versus all tropical and subtropical moist broad-leaved forest (less than 1.5%), their contribution to global CH_4_ uptake is negligible.

#### Estimating the CO_2_ equivalence of methane uptake and comparisons with ecosystem C dynamics

To examine the relative importance of CH_4_ uptake, we compared it to the C fluxes and stocks of forests. Although metrics such as global warming potential (GWP) have long been used, a recent consensus has developed that expressing CH_4_ emissions as CO_2_ equivalent emissions using GWP-100 overstates the effect of constant CH_4_ emissions on global surface temperature by a factor of 3–4 (ref. ^52^) while understating the effect of any new CH_4_ source or sink by a factor of 4–5 over the 20 years following the introduction of the new source (IPCC AR6). A more accurate indication of CO_2_-we emissions is to equate a constant 1 t yr^−1^ of CH_4_ source that is more than 20 years old with 8 t of CO_2_-we yr^−1^ but to account for the warming (or cooling) impact of a 1 t of CH_4_ yr^−1^ step-change in CH_4_ emission rate by adding (or subtracting) 120 t of CO_2_-we yr^−1^ over the 20 years following the change. Hence, a new constant source (or sink) of CH_4_ introduced in year 1 is equated with 128 t of CO_2_-we for years 1–20 and 8 t of CO_2_-we thereafter^53^. For direct comparisons with ecosystem C stocks and fluxes, we converted all CO_2_-we values to C only.

#### Effects of changing forest area analysis

Tropical deforestation entails the sudden loss of this woody surface CH_4_ sink, corresponding to a net loss of 128 t of CO_2_-we per t of CH_4_ for the first 20 years following deforestation (Methods) or a total ‘stock loss’ of 2,560(20 × 128) t of CO_2_-we per t of CH_4_ yr^−1^ of the CH_4_ sink. Tropical forest was lost at a rate of 10.3 million ha yr^−1^ over the period 2002–2018 (ref. ^54^). This results in 0.59 Mg of CO_2_-we-C ha^−1^ yr^−1^ sink reduction (2.15 Mg of CO_2_-we ha^−1^ yr^−1^) or a total of 6.04 Tg of CO_2_-we-C from the act of deforestation, a small extra climate impact, dwarfed by the release of biomass C stocks (1 Pg of C yr^−1^). It remains unknown how quickly a methanotrophic community equivalent to that of a mature forest takes to develop, but, if it develops quickly, the CH_4_ sink in young secondary forests is likely to be similar to that in mature forests. Hence, the CH_4_ sink benefits of new forest could manifest much more quickly than the C storage benefits. Assuming a similar woody surface area and CH_4_ sink per hectare as for mature forests and a CO_2_-we of 128 for the first 20 year timeframe of interest, the tree CH_4_ sink would add an extra greenhouse gas mitigation value of 0.131 and 0.586 Mg of CO_2_-we-C ha^−1^ yr^−1^ in temperate and tropical forests, respectively, corresponding to a 7% and 12% extra climate benefit of new trees in these respective biomes.

## Online content

Any methods, additional references, Nature Portfolio reporting summaries, source data, extended data, supplementary information, acknowledgements, peer review information; details of author contributions and competing interests; and statements of data and code availability are available at 10.1038/s41586-024-07592-w.

### Supplementary information

Supplementary Information Supplementary Table 1, acknowledgements and references.

### Source data

Source Data Fig. 1

Source Data Fig. 2

Source Data Fig. 3

## Figures and Tables

**Extended Data Table 1:**
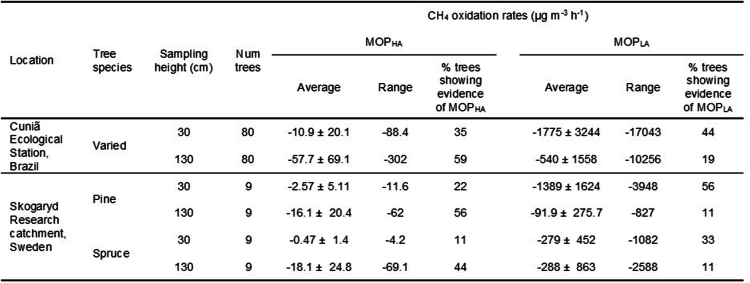
Wood core methane oxidation potentials

High-affinity (MOP_HA_) and low-affinity methane (CH_4_) oxidation potentials (MOP_LA_) measured from wood cores extracted at 30 and 130 cm stem height at Cuniã, Brazil and Skogaryd, Sweden forest sites. MOP_LA_ tended to be higher in tree stem bases than in the uppermost woody surface sampling position with higher percentages of trees demonstrating MOP_LA_ at 30 cm (44% for Cunia and 56% and 33% for pine and spruce respectively) than at 130 cm (19% for Cunia and 11% for both pine and spruce) across all biomes and species. By contrast, the situation was reversed for MOP_HA_, with higher percentages of trees demonstrating MOP_HA_ at 130 cm (59% for Cunia and 56% and 44% for pine and spruce respectively) than at 30 cm (35% for Cunia and 22% and 11% for pine and spruce respectively) and with higher MOP_HA_ at 130 cm.

**Extended Data Table 2:**
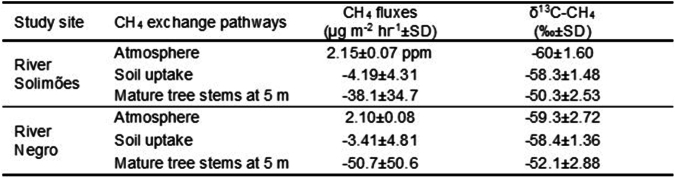
Stable carbon isotope values of CH_4_ within flux chambers on woody and soil surface

Atmospheric CH_4_ concentrations in ppm were measured by drawing atmospheric air next to the tree under investigation, at 1.3 m above the soil surface, using a syringe. A second syringe sample, extracted at the same time, was used to analyse δ^13^C-CH_4_ in atmospheric air. Stem and soil CH_4_ flux were measured in real time using a 10-minute chamber closure time. At the end of the flux measurement, gas samples were extracted for δ^13^C-CH_4_ analysis. N = 36 for stem and soil CH_4_ flux and δ^13^C-CH_4_ analysis at each location.

**Extended Data Table 3:**
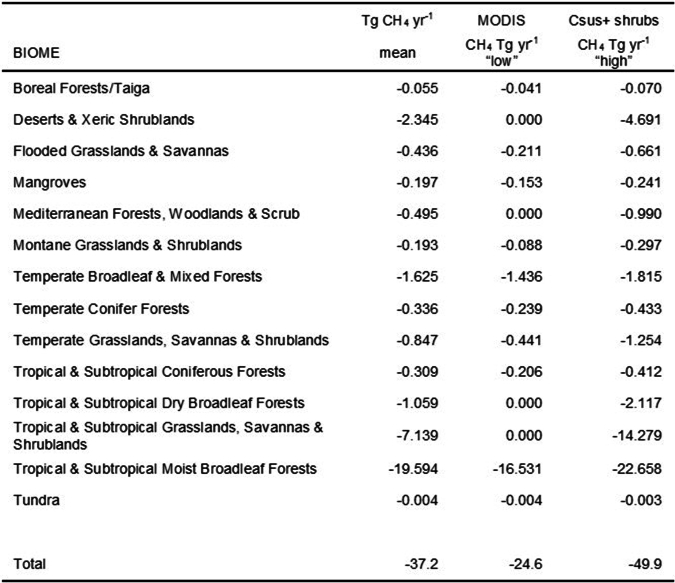
Mean and high/low estimates of total annual CH_4_ uptake per biome

The low estimate uses the MODIS VCF product and the high estimate uses the 1 km global consensus land cover project which includes woody shrubs. We zeroed uptake in probable water-limited biomes in our low estimate in keeping with our conservative approach. Parameters used for the different estimates are presented in Extended Data Table 4. See Methods.

**Extended Data Table 4:**
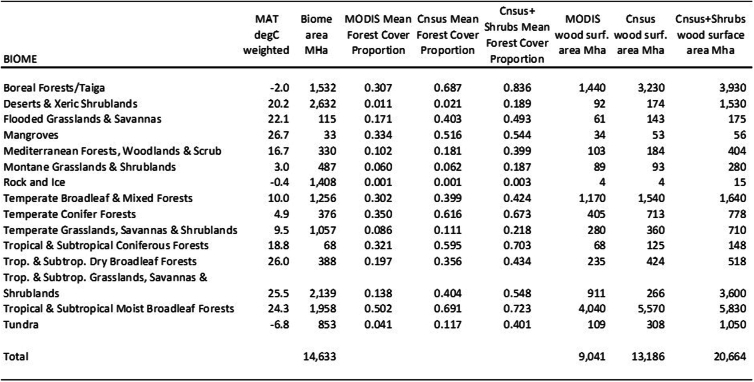
Parameters employed to estimate total annual forest biome methane uptake

*MAT weighted* is mean annual temperature across the biome weighted by forest cover in each pixel. *Biome area* is the total land area in that biome. We applied a woody area index (WAI) of 4.12 for tropical and subtropical moist broad-leaved forest and 3.07 for remaining biomes from our revised forest allometry. Forest cover proportion is derived from the two land cover products we use. The consensus land cover product includes shrub cover as a separate category, so we calculate woody surface area with and without them. We assign the mean tropical forest woody surface area per ground area value to shrubs.

**Extended Data Table 5:**
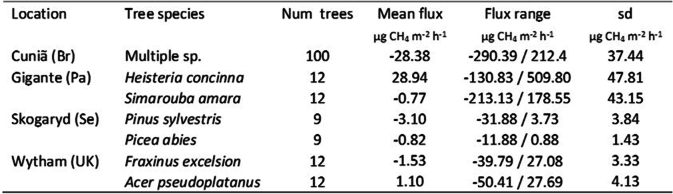
General information of study sites

Mean flux and standard deviation (sd) are calculated from the mean fluxes from each tree after averaging fluxes from different sampling positions above the forest floor. Flux ranges are the maximum and minimum fluxes from any stem height for each location and species.

**Extended Data Table 6:**
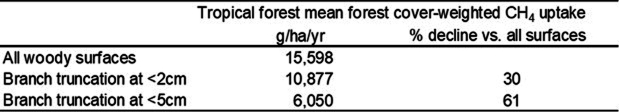
Branch size contribution to woody surface uptake in tropical forests

Table shows how different branch sizes contribute to total woody surface methane uptake in tropical forests.

## Data Availability

Methane flux data from all sites included in our analysis are included both in the data files associated with each figure as well as on the UBIRA e-Data repository at 10.25500/edata.bham.00001060. Source data are provided with this paper.
