# Supplementary material for: Global atmospheric methane uptake by upland tree woody surfaces
Source: Nature. 2024 Jul 24;631(8022):796–800. doi: 10.1038/s41586-024-07592-w (PMC11269171; doi:10.1038/s41586-024-07592-w)
Supplement: Supplementary file 1 — Supplementary Table 1, acknowledgements and references. [file 41586_2024_7592_MOESM1_ESM.pdf]

nature portfolio
https://doi.org/10.1038/s41586-024-07592-w

Supplementary information

Global atmospheric methane uptake by upland tree woody surfaces

In the format provided by the authors and unedited

Supplementary Information Guide.

Supplementary Table. Table S1. Summary of studies measuring tree stem CH_4 fluxes in upland (free-drained soil) boreal, temperate and tropical ecosystems. Page 1

Supplementary Acknowledgments. Acknowledgements for CTFS/ForestGeo site data use. Page 2.

Supplementary References. Page 4

Table S1

Summary of studies measuring tree stem CH_4 fluxes in upland (free-drained soil) boreal, temperate and tropical ecosystems. Most of the studies reporting mean net emissions also presented CH_4 uptake by tree stems, but this is rarely discussed.

Reference	Climatic region	Reported uptake	Discussed uptake	Measurement height	Tree species	Num of trees	Notes
This study	Boreal, Temperate, Tropical	**Yes**	Yes	20, 30, 60, 75, 100, 130, 140, 180, 200 cm*	Multiple	274	Not all stem heights were measured in all trees and locations
Sundqvist et al., 2012	Boreal	**Yes**	Yes	NA	Multiple (4 species)	7	Fluxes measured on branches with leaves<br>Mean flux: -10.9 μg m^{-2} h^{-1}
Machacova et al., 2016	Boreal	**Yes**	No	20 cm	*Pinus sylvestris*	12	
Wang et al., 2016	Temperate	No	-	50, 130, 230, 450 cm	Multiple (3 species)	NA	
Pitz et al., 2017	Temperate	**Yes**	No	45 cm	Multiple (7 species)	17	Mean uptake: -1.09 μg m^{-2} h^{-1}<br>9% of measurements were uptake
Warner et al., 2017	Temperate	**Yes**	No	140 cm	Multiple (6 species)	16	
Wang et al., 2017	Temperate	No	-	150cm	*Populus davidiana*	15	
Welch et al., 2018	Tropical	**Yes**	No	30 cm	Multiple (2 species)	24	
Pitz et al., 2018	Temperate	**Yes**	No	45 cm	Multiple (7 species)	19	
Maier et al., 2018	Temperate	No	-	40, 120, 200 cm	*Fagus sylvatica*	10	
Plain et al., 2019	Temperate	No	-	25 cm	*Quercus petraea*	3	
Barba et al., 2019	Temperate	**Yes**	No	75, 150 cm	*Carya cordiformis*	1	
Vainio., 2019	Boreal	**Yes**	No	20 - 730 cm	Multiple (3 species)	17	
Schindler et al., 2020	Temperate	No	-	0, 80, 170 cm	*Alnus incana*	13	
Flanagan et al., 2020	Temperate	No	-	50 cm	Multiple (2 species)	5	
Iddris, 2020	Tropical	No	-	130 cm	Multiple (NA)	144	
Megonigal et al., 2020	Temperate	No	-	NA	Multiple (3 species)	11	
Machacova et al., 2020	Tropical	**Yes**	Yes	40, 110, 182 cm	Multiple (6 species)	24	Mean flux: -15.6 ± 2.02 μg m^{-2} h^{-1}
Barba et al., 2021	Temperate	**Yes**	No	50, 100, 150 cm	*Carya cordiformis*	18	20% of measurements were uptake
Moldaschl et al., 2021	Temperate	**Yes**	No	30, 160, 360 cm	*Fraxinus excelsior*	6	Flux range: -1.08 to 1.34 μg m^{-2} h^{-1} (non-flooded plots)
Plain et al., 2021	Temperate	No	-	20 cm	Multiple (2 species)	2	
Bréchet et al., 2021	Tropical	**Yes**	No	130 cm	*Eperua falcata*	1	
Feng et al., 2022	Subtropical	No	na	50, 100, 150 cm	*Populus deltoides x P. euramericana*	12	
Han et al. 2022	Subtropical / temperate	No	na	50, 100, 150 cm	Multiple (2 species)	24	Flux range: 1.12 to 1357.01 μg m^{-2} h^{-1}
Warren et al., 2022	Tropical	**Yes**	No	130 cm	Multiple	20	Median flux and quartiles: -4.08 (11.76, -13.48) μg m^{-2} h^{-1}
Vainio et al., 2022	Boreal	**Yes**	No	30, 350, 750 cm	*Betula pubescens*	1	
Epron et., 2023	Temperate	No	na	80, 160, 350 cm	Multiple (5 species)	10	Flux range: 0 to 13.32 μg m^{-2} h^{-1}
Machacova et al., 2023	Temperate	**Yes**	No	40, 120, 200 cm	*Fagus sylvatica*	20	Flux range: −4.37 and 173.97 μg m^{-2} h^{-1}

Supplementary Acknowledgements

The 50-ha Wanang Forest Dynamics Plot is a collaborative project of the New Guinea Binatang Research Center, the Center for Tropical Forest Science of the Smithsonian Tropical Research Institute, the Forest Research Institute of Papua New Guinea, the Czech Academy of Sciences and the University of Minnesota supported by NSF DEB- 1027297 and NIH ICBG 5UO1TW006671. We acknowledge the government of Papua New Guinea and the customary landowners of Wanang for supporting and maintaining the plot (Vincent et al., 2015).

This research was supported by grants BSR-8811902, DEB 9411973, DEB 0080538, DEB 0218039, DEB 0620910, DEB 0963447 AND DEB-129764 from NSF to the Department of Environmental Science, University of Puerto Rico, and to the International Institute of Tropical Forestry, USDA Forest Service, as part of the Luquillo Long-Term Ecological Research Program. The U.S. Forest Service (Dept. of Agriculture) and the University of Puerto Rico gave additional support. The LFDP is part of the Smithsonian Institution Forest Global Earth Observatory, a worldwide network of large, long-term forest dynamics plots. (Zimmerman et al., 2010)

Data from the SERC ForestGeo plot were provided by Geoffrey Parker on Dec 4, 2019. These data were gathered as part of forest ecology studies at the Smithsonian Environmental Research Center (SERC). SERC is a participant in the Smithsonian Institution Forest Global Earth Observatory (ForestGEO) network (Holmes, 2012).

The UCSC Forest Ecology Research Plot was made possible by National Science Foundation grants to Gregory S. Gilbert (DEB-0515520 and DEB-084259), by the Pepper-Giberson Chair Fund, the University of California, and the hard work of dozens of UCSC students. The plot project is part the Center for Tropical Forest Science, a global network of large-scale demographic tree plots (Gilbert et al., 2010).

Funding for the Smithsonian Conservation Biology Institute (SCBI) Large Forest Dynamics Plot (LFDP) was provided by the Smithsonian Institution, the National Zoological Park, and the HSBC Climate Partnership. The SCBI LFDP is part of the Smithsonian Institution Forest Global Earth Observatory, a worldwide network of large, long-term forest dynamics plots (Bourg et al., 2013).

The BCI forest dynamics research project was made possible by National Science Foundation grants to Stephen P. Hubbell: DEB-0640386, DEB-0425651, DEB-0346488, DEB-0129874, DEB-00753102, DEB-9909347, DEB-9615226, DEB-9615226, DEB-9405933, DEB-9221033, DEB-9100058, DEB-8906869, DEB-8605042, DEB-8206992, DEB-7922197, support from the Center for Tropical Forest Science-Forest Global Earth Observatory, the Smithsonian Tropical Research Institute, the John D. and Catherine T. MacArthur Foundation, the Mellon Foundation, the Small World Institute Fund, and numerous private individuals, and through the hard work of over 100 people from 10 countries over the past three decades. The plot project is part the Center for Tropical Forest Science-Forest Global Earth Observatory (CTFS-ForestGEO), a global network of large-scale demographic tree plots (Condit et al., 2019; Condit et al., 1998; Hubbell et al., 1999; Condit et al., 2019b).

The 25-ha Long-Term Ecological Research Project at Sinharaja World Heritage Site is a collaborative project of the Uva Wellassa University, the University of Peradeniya, the Center for Tropical Forest Science of the Smithsonian Tropical Research Institute and the Arnold Arboretum of Harvard

University, USA, with supplementary funding received from the John D. and Catherine T. Macarthur Foundation, the National Institute for Environmental Science, Japan, and the Helmholtz Centre for Environmental Research-UFZ, Germany, for past censuses. The PIs gratefully acknowledge the Forest Department and the Post-Graduate Institute of Science at the University of Peradeniya, Sri Lanka for supporting this project , and the local field and lab staff who tirelessly contributed in the repeated censuses of this plot.

Al-Amin Iddris N. **2020**. Trace gas fluxes from soils and tree stems of rainforests and cacao agroforests in the Congo Basin, Cameroon.

Barba J, Poyatos R, Capooci M, Vargas R. **2021**. Spatiotemporal variability and origin of CO2 and CH4 tree stem fluxes in an upland forest. *Global Change Biology*: gcb.15783.

Barba J, Poyatos R, Vargas R. **2019**. Automated measurements of greenhouse gases fluxes from tree stems and soils: magnitudes, patterns and drivers. *Scientific Reports* **9**: 4005.

Bourg, N.A., W.J. McShea, J.R. Thompson, J.C. McGarvey, and X. Shen. 2013. Initial census, woody seedling, seed rain, and stand structure data for the SCBI SIGEO Large Forest Dynamics Plot. Ecology 94(9): 2111-2112. http://dx.doi.org/10.1890/13-0010.1

Bréchet LM, Daniel W, Stahl C, Burban B, Goret J-Y, Salomón RL, Janssens IA. **2021**. Simultaneous tree stem and soil greenhouse gas (CO2, CH4, N2O) flux measurements: a novel design for continuous monitoring towards improving flux estimates and temporal resolution. *New Phytologist* **230**: 2487–2500.

Condit R., Perez, R., Aguilar, S., Lao, S., Foster, R., Hubbell, S.P. 2019. Complete data from the Barro Colorado 50-ha plot: 423617 trees, 35 years, 2019 version.

Condit, R. 1998. Tropical Forest Census Plots. Springer-Verlag and R. G. Landes Company, Berlin, Germany, and Georgetown, Texas.

Condit R., Perez, R., Aguilar, S., Lao, S., Foster, R., Hubbell, S.P. 2019b. BCI 50-ha plot taxonomy, 2019 version.

Daniel W, Stahl C, Burban B, Goret JY, Cazal J, Richter A, Janssens IA, Bréchet LM. **2023**. Tree stem and soil methane and nitrous oxide fluxes, but not carbon dioxide fluxes, switch sign along a topographic gradient in a tropical forest. *Plant and Soil* **488**: 533–549.

Epron D, Mochidome T, Bassar ATMZ, Suwa R. **2023**. Variability in methane emissions from stems and buttress roots of Bruguiera gymnorrhiza trees in a subtropical mangrove forest. *Ecological Research*. DOI: 10.1111/1440-1703.12415

Feng H, Guo J, Ma X, Han M, Kneeshaw D, Sun H, Malghani S, Chen H, Wang W. **2022**. Methane emissions may be driven by hydrogenotrophic methanogens inhabiting the stem tissues of poplar. *New Phytologist* **233**: 182–193.

Flanagan L, Nikkel D, Scherloski L, Tkach R, Smits K, Selinger L, Rood S. **2021**. Multiple processes contribute to methane emission in a riparian cottonwood forest ecosystem. *New Phytologist* **229**: 1970–1982.

Gilbert, G.S., E. Howard, B. Ayala-Orozco, M. Bonilla-Moheno, J. Cummings, S. Langridge, I.M. Parker, J. Pasari, D. Schweizer, S. Swope. 2010. Beyond the tropics: forest structure in a temperate forest mapped plot. Journal of Vegetation Science 21: 388-405.

Han M, Feng H, Peng C, Lei X, Xue J, Malghani S, Ma X, Song X, Wang W. **2022**. Spatiotemporal patterns and drivers of stem methane flux from two poplar forests with different soil textures. *Tree Physiology* **42**: 2454–2467.

Holmes, R.T. 2012. Bird Abundances at Hubbard Brook (1969-2010) and on three replicate plots (1986-2000) in the White Mountain National Forest. Durham, NH. Hubbard Brook Data Archive [Database]. http://hubbardbrook.org/data/dataset.php?id=81 (23 July 2012)

Hubbell, S.P., R.B. Foster, S.T. O'Brien, K.E. Harms, R. Condit, B. Wechsler, S.J. Wright, and S. Loo

de Lao. 1999. Light gap disturbances, recruitment limitation, and tree diversity in a neotropical forest. Science 283: 554-557.

Machacova K, Bäck J, Vanhatalo A, Halmeenmäki E, Kolari P, Mammarella I, Pumpanen J, Acosta M, Urban O, Pihlatie M. **2016**. Pinus sylvestris as a missing source of nitrous oxide and methane in boreal forest. *Scientific Reports* **6**: 23410.

Machacova K, Borak L, Agyei T, Schindler T, Soosaar K, Mander Ü, Ah-Peng C. **2021**. Trees as net sinks for methane (CH 4) and nitrous oxide (N 2 O) in the lowland tropical rain forest on volcanic Réunion Island. *New Phytologist* **229**: 1983–1994.

Machacova K, Warlo H, Svobodová K, Agyei T, Uchytilová T, Horáček P, Lang F. **2023**. Methane emission from stems of European beech (Fagus sylvatica) offsets as much as half of methane oxidation in soil. *New Phytologist* **238**: 584–597.

Maier M, Machacova K, Lang F, Svobodova K, Urban O. **2018**. Combining soil and tree-stem flux measurements and soil gas profiles to understand CH 4 pathways in Fagus sylvatica forests. *Journal of Plant Nutrition and Soil Science* **181**: 31–35.

Megonigal JP, Brewer PE, Knee KL. **2020**. Radon as a natural tracer of gas transport through trees. *New Phytologist* **225**: 1470–1475.

Moldaschl E, Kitzler B, Machacova K, Schindler T, Schindlbacher A. **2021**. Stem CH4 and N2O fluxes of Fraxinus excelsior and Populus alba trees along a flooding gradient. *Plant and Soil 2021 461:1* **461**: 407–420.

Pitz S, Megonigal JP. **2017**. Temperate forest methane sink diminished by tree emissions. *New Phytologist* **214**: 1432–1439.

Pitz S, Megonigal JP, Chang C-H, Szlavecz K. **2018**. Methane fluxes from tree stems and soils along a habitat gradient. *Biogeochemistry* **137**: 307–320.

Plain C, Epron D. **2021**. Pulse labelling of deep soil layers in forest with 13 CH 4 : testing a new method for tracing methane in the upper horizons, understorey vegetation and tree stems using laser-based spectrometry. *Biogeochemistry* **153**: 215–222.

Plain C, Ndiaye F-K, Bonnaud P, Ranger J, Epron D. **2019**. Impact of vegetation on the methane budget of a temperate forest. *New Phytologist* **221**: 1447–1456.

Schindler T, Mander Ü, Machacova K, Espenberg M, Krasnov D, Escuer-Gatius J, Veber G, Pärn J, Soosaar K. **2020**. Short-term flooding increases CH4 and N2O emissions from trees in a riparian forest soil-stem continuum. *Scientific reports* **10**: 3204.

Sundqvist E, Crill P, Mölder M, Vestin P, Lindroth A. **2012**. Atmospheric methane removal by boreal plants. *Geophysical Research Letters* **39**: 1–6.

Vainio E. **2019**. The Contributions of Soil, Ground Vegetation and Trees to the Methane Exchange of Boreal Forest.

Vainio E, Haikarainen IP, Machacova K, Putkinen A, Santalahti M, Koskinen M, Fritze H, Tuomivirta T, Pihlatie M, Vainio E, *et al.* **2022**. Soil-tree-atmosphere CH 4 flux dynamics of boreal birch and spruce trees during spring leaf-out. *Plant and Soil*: (in press).

Vincent J.B., Henning B., Saulei S., Sosanika G., & Weiblen G.D. 2015. Forest carbon in lowland Papua New Guinea: local variation and the importance of small trees. Austral Ecology. 40:151-159.

Wang Z-P, Gu Q, Deng F-D, Huang J-H, Megonigal JP, Yu Q, Lu X-T, Li L-H, Chan S, Zhang Y-H, *et al.* **2016**. Methane emissions from the trunks of living trees on upland soils. *New Phytologist* **211**: 429–

439.

Wang Z-P, Han S-J, Li H-L, Deng F-D, Zheng Y-H, Liu H-F, Han X-G. **2017**. Methane production explained largely by water content in the heartwood of living trees in upland forests. *Journal of Geophysical Research: Biogeosciences* **122**: 2479–2489.

Warner DL, Villarreal S, McWilliams K, Inamdar S, Vargas R. **2017**. Carbon dioxide and methane fluxes from tree stems, coarse woody debris, and soils in an upland temperate forest. *Ecosystems* **20**: 1205–1216.

Welch B, Gauci V, Sayer EJ. **2019**. Tree stem bases are sources of CH4 and N2O in a tropical forest on upland soil during the dry to wet season transition. *Global Change Biology* **25**: 361–372.

Zimmerman, Jess K., Liza S. Comita, Jill Thompson, María Uriarte, and Nicholas Brokaw. 2010. Patch dynamics and community metastability of a subtropical forest: compound effects of natural disturbance and human land use. Landscape Ecology 25: 1099-1111.
